# A prospective study on dual time ^18^F-FDG-PET/CT in high-risk prostate cancer patients

**DOI:** 10.1186/s13104-018-3985-2

**Published:** 2018-12-07

**Authors:** Mike Allan Mortensen, Mie Holm Vilstrup, Mads Hvid Poulsen, Oke Gerke, Poul Flemming Høilund-Carlsen, Lars Lund

**Affiliations:** 1 Department of Urology, Odense University Hospital, Odense, Denmark; 2 Department of Clinical Research, University of Southern Denmark, Odense, Denmark; 3 Department of Nuclear Medicine, Odense University Hospital, Odense, Denmark; 4 Academy of Geriatric Cancer Research (AgeCare), Odense University Hospital, Odense, Denmark

**Keywords:** Prostatic neoplasms, Diagnostic imaging, Positron emission tomography, Fluorodeoxyglucose

## Abstract

**Objective:**

This proof of concept study investigated whether dual time point FDG-PET/CT with image acquisition after 1 and 3 h could be useful in preoperative staging of patients undergoing robot-assisted radical prostatectomy and extended pelvic lymph node dissection for high-risk prostate cancer.

**Results:**

Twenty patients with high-risk prostate cancer underwent dual time point FDG-PET/CT before undergoing surgery. Histologically confirmed lymph node metastases were found in 9/20 (45%). A median of 19 (range 10–41; n = 434) lymph nodes were removed per patient. Pelvic lymph nodes with detectable FDG uptake were seen in two patients only, but the FDG-avid lesion on PET did not correspond with pathological findings in either patient. We found a significant increase in maximal standardized uptake value of the prostate of around 30% between early and late imaging. We found no correlation between clinical findings after radical prostatectomy and PET measurements.

## Introduction

Using ^18^F-fluorodeoxyglucose (FDG) in combination with positron emission tomography/computed tomography (PET/CT) in prostate cancer management is controversial [1, 2]. Malignant cells in general have higher glucose metabolism and hence higher FDG-uptake than benign cells [3]. Although the same is true for prostate cancer, FDG uptake is generally low in prostate cancer cells, often causing tracer uptake to be so small that segregation between malign and benign tissue is not possible [3, 4]. Studies on the use of FDG-PET/CT in newly diagnosed prostate cancer are few and have focused primarily on staging of bone metastases [5–7].

In most FDG-PET studies, images are acquired 60 min after injection. This time point is entirely arbitrary. Studies suggest that FDG uptake takes place over several hours in malignant cells and that later image acquisition may therefore be more useful [8]. In tumours with low FDG uptake, postponing image acquisition could be even more beneficial, allowing for tumour-to-background differentiation otherwise not possible [9]. For these reasons, several studies conducted in diverse settings have tested the use of dual time point FDG-PET/CT in the breast, liver and lung, suggesting that dual or late imaging might increase both sensitivity and specificity in detecting cancerous lesions [10]. It has been indicated that the percentage change between early and late imaging as expressed by a retention index (RI) correlates with the disease stage and aggressiveness [11–13].

The use of dual time point FDG-PET/CT in staging of prostate cancer has never been investigated in a prospective setting [14].

The aim of this proof-of-concept study was to evaluate the usefulness of dual time point FDG-PET/CT in patients undergoing robot-assisted radical prostatectomy (RARP) with extended pelvic lymph node dissection (ePLND).

## Main text

### Materials and methods

This prospective single centre proof-of-concept study was conducted between January 2015 and September 2017. Patients with high-risk prostate cancer according to D’Amico criteria underwent dual time point FDG-PET/CT before RARP with ePLND or ePLND alone before external beam radiation [15]. Patients with other known malignancies and patients with diabetes were not offered inclusion.

### Imaging protocol

Patients were required to fast for a minimum of 6 h before the FDG-PET/CT scan. Blood glucose level was determined before tracer injection (max 150 mg/dL). FDG was administered intravenously in a dose of 4 MBq/kg (max 400 MBq). The initial scan was performed after 60 min; the second after 180 min. All patients underwent diagnostic contrast-enhanced CT either as part of the procedure or as part of other imaging.

FDG-PET/CT scans were performed on either GE Discovery RX or Discovery STE (GE Medical Systems, Milwaukee, WI) integrated PET/CT scanners.

### Image interpretation

Early and late FDG scans were interpreted by an experienced nuclear medicine specialist (MHV) blinded to histopathological results. Pelvic lymph node regions and the prostate were evaluated using a visual likelihood scale ranging from 0 to 4 (0 = no uptake, 1 = almost certainly benign, 2 = probably benign, 3 = probably malign, 4 = almost certainly malign) on both early and late scans. Maximal standardized uptake values (SUVmax) of the prostate were calculated for both the early and the late scan. In order to reflect change between early and late imaging, RI (%) was calculated by subtracting the SUVmax at 60 min from the SUVmax at 180 min and dividing by SUVmax at 60 min.

### Surgical procedure

All patients underwent ePLND performed either laparoscopically as a stand-alone procedure or during RARP in concordance with European guidelines [16]. Removed lymph nodes were mapped meticulously according to the region from which they were removed-along the external iliac artery, along the internal iliac artery and in the obturator fossa bilaterally.

### Histological examination

Pre-operative core biopsies of the prostate were processed according to department procedure with description of extension in each core and Gleason Score.

Prostatectomy specimens were all processed according to routine department procedures and in accordance with recommendations from the International Society of Urological Pathology [17]. Specimen weight after removal of the seminal vesicles was recorded. Gleason Score, pathological tumour stage, surgical margins and tumour extension were evaluated. Based on the estimated extension and prostate weight, an approximate tumour weight was calculated.

### Statistics

Patient demographics were analysed using descriptive statistics. Early and late imaging results were compared using Student’s paired t-test. Correlation between findings on dual time point FDG-PET/CT and clinical features was tested using Spearman’s rank correlation coefficient. All analyses were performed using STATA/IC 15.1 (StataCorp, College Station, Texas, USA).

### Results

Twenty high-risk patients with a median age of 67 years (range 53–75) were included in the study. Median prostate-specific antigen (PSA) was 17 (range 1.4–35). A Gleason Score of 4 + 3 or higher was seen in 15/20 patients (75%). Locally advanced disease was suspected in 7/20 patients (35%). More detailed characteristics of the included patients can be seen in Table 1.

**Table 1:** Patient characteristics

PSA (ng/mL)	
Median (range)	17.0 (1.4–35.0)
No. Gleason score (%)	
≤ 3 + 4	5 (25%)
≥ 4 + 3	15 (75%)
No. clinical stage (%)	
cT1	5 (25%)
cT2	8 (40%)
cT3	7 (35%)

Two patients (10%) underwent ePLND as a stand-alone procedure; 18 patients (90%) underwent ePLND as part of RARP. Post-operative tumour characteristics after RARP can be seen in Table 2.

**Table 2:** Post-operative tumour characteristics

No. Gleason score (%)	
3 + 4	6 (33%)
4 + 3	7 (39%)
≥ 4 + 4	5 (28%)
No. pathological stage (%)	
pT2c	8 (44%)
pT3a	7 (39%)
pT3b	3 (17%)
Estimated tumour weight (g)	
Mean (± SD)	10.3 (± 4.7)

A total of 434 lymph nodes were removed. The median number of lymph nodes removed per patient was 19 (range 10–41). Histologically confirmed lymph node metastases were found in nine patients (45%) with a total of 13 malignant lymph nodes. The size of metastatic lymph nodes ranged from 2 to 16 mm. In one patient, mantle cell lymphoma was detected alongside a metastasis from prostate cancer.

All but one patient underwent surgical intervention within 2 months of dual time point FDG-PET/CT. Median time from imaging to surgery was 15 days (range 3–65 days). Median time to early and late scan was 63 min (range 58–81) and 181 min (range 178–199), respectively.

Pelvic lymph nodes with detectable FDG-uptake were seen in two patients only (visual likelihood score 1). No change in visual likelihood score was seen between early and late imaging. Both patients were diagnosed with lymph node metastatic disease after lymph node dissection, but the FDG-avid lesion on PET did not correspond with pathological findings in either patient. Lymph node regions of interest were not described as more easily identifiable on late than on early imaging.

Despite histopathologically confirmed tumour in the prostate, a visual likelihood score of 3 or 4 (“probably malign” and “almost certainly malign”) was seen in only 5/20 patients on early imaging compared with 9/20 patients in late imaging. SUVmax increased significantly between early and late scans with a median SUVmax of 5.7 (range 2.2–19.1) and 7.5 (range 2.2–19.8) in early and late scans, respectively (p < 0.001). Median RI was 32% (range 0–152%).

We found no correlation between clinical findings after radical prostatectomy (PSA, Gleason score pathological T-stage and tumour volume) and SUVmax (early or late) or RI.

### Discussion

We found that dual time FDG-PET/CT did not aid detection of lymph node metastases in this high-risk population even if nearly half of the patients had lymph node metastases at the time of surgery. Only two patients had lymph nodes with FDG uptake, both with minimal uptake and no change from early to late imaging. Both patients had metastatic lymph nodes confirmed by histopathological examination. Still, in both cases, the malignant lymph node was located in a different lymph node region, leaving no doubt that the seen lymph nodes and the actual malignant lymph nodes were not the same.

Uptake in the prostate was significantly higher in late than in early imaging, with a median increase in SUVmax exceeding 30%. This finding supports the hypothesis of continuous tracer uptake after 1 h in malignant lesions.

A previous study on 28 patients with untreated prostate cancer suggested that lesions with higher FDG uptake in the prostate may be more aggressive and that high FDG uptake in the prostate would be found predominantly in patients with disseminated disease [18]. In the present small population, we found no correlation between uptake values in the prostate and clinical characteristics, including the presence of lymph node metastases. Although all included patients were diagnosed with prostate cancer, most had no focal cancer-suspicious tracer uptake at either early or late imaging. A recent study on dual time FDG-PET/CT in staging of 35 patients with squamous cell cancer of the oesophagus found that the RI of a given lymph node lesion best reflected whether the lesion was malignant or not [13]. In contrast, in breast cancer, dual imaging gave no additional information compared with early imaging only [19]. As dual time point FDG-PET/CT was not able to identify malignant lymph nodes at all in our study, such calculations could not be made. With RI assumed to be a marker for tumour metabolism, one would expect that a correlation between RI and tumour characteristics could be found. We found no such correlation between RI and histopathological features of the prostatic tumour.

The evident null-results in this study combined with the previous discouraging studies on conventional FDG-PET/CT suggests that FDG-PET/CT has no role in the management of patients with prostate cancer. In recent years, molecular imaging with tracers targeting prostate-specific membrane antigen (PSMA) has emerged as a promising tool for prostate cancer imaging [20]. We believe that future studies on molecular imaging in staging of prostate cancer should focus on validating this novel imaging modality as larger, prospective studies are still warranted.

### Conclusion

Dual time point FDG-PET/CT is not a useful tool for preoperative staging in patients with high-risk prostate cancer. Neither usual FDG-PET/CT (early imaging) nor late or dual time point imaging gave useful information about the lymph node status or the aggressiveness of the tumour in patients in this study.

## Limitations

This study is limited by its small size and its homogenous population. Also, although nearly half of the patients had lymph node metastatic disease, only a few and rather small malignant lymph nodes were found per patient.

## Abbreviations

ePLND: extended pelvic lymph node dissection
FDG: ^18^F-fluorodeoxyglucose
PET/CT: positron emission tomography/computed tomography
PSA: prostate-specific antigen
RARP: robot-assisted radical prostatectomy
RI: retention index
SUVmax: maximal standardized uptake value
